# Chemosensory anxiety cues moderate the experience of social exclusion – an fMRI investigation with Cyberball

**DOI:** 10.3389/fpsyg.2015.01475

**Published:** 2015-10-09

**Authors:** Olga A. Wudarczyk, Nils Kohn, Rene Bergs, Raquel E. Gur, Bruce Turetsky, Frank Schneider, Ute Habel

**Affiliations:** ^1^Department of Psychiatry, Psychotherapy and Psychosomatics, Faculty of Medicine, RWTH Aachen UniversityAachen, Germany; ^2^JARA – Translational Brain Medicine, RWTH Aachen University and Research Centre JülichAachen, Germany; ^3^Department for Cognitive Neuroscience, Donders Institute for Brain, Cognition and Behaviour, Radboud University Nijmegen Medical CentreNijmegen, Netherlands; ^4^Department of Psychiatry, University of PennsylvaniaPhiladelphia, PA, USA; ^5^Institute of Neuroscience and Medicine (INM-6), Jülich Research CentreJülich, Germany

**Keywords:** social exclusion, chemosignals, cyberball, ostracism, olfaction, anxiety

## Abstract

Recent evidence suggests that the experience of stress can be communicated between individuals via chemosensory cues. Little is known, however, about the impact of these cues on neurophysiological responses during a socially threatening situation. In the current investigation we implemented a widely used paradigm to study social exclusion—Cyberball—to examine whether chemosensory cues signaling anxiety modulate the neuronal effects of ostracism. In a double-blind, within-subjects design, 24 healthy, normosmic participants were presented with chemosensory cues of anxiety (or control samples) and completed the Cyberball task while in a 3T fMRI scanner. Axillary sweat collected from male students awaiting an oral examination served as the anxiety cues while the chemosensory control stimuli consisted of sweat collected from the same individuals participating in an ergometer training session. The neuroimaging data revealed that under the control chemosensory condition, exclusion from Cyberball was associated with significantly higher orbitofrontal cortex and anterior cingulate cortex activity, which is consistent with previous studies in the field. However, when participants were primed with the anxiety sweat, the activity in these regions was not observed. Further, under exposure to anxiety cues during ostracism the participants showed deactivations in brain regions involved in memory (hippocampus), social cognition (middle temporal gyrus, superior temporal gyrus) and processing of salience (inferior frontal gyrus). These results suggest that successful communication of anxiety via the chemosensory domain may moderate the experience of social exclusion. It is possible that the anxiety signals make it easier for the individuals to detach from the group, pointing to the communicative role of chemosensory anxiety cues in enhancing adjustment mechanisms in light of a distressing situation.

## Introduction

Recent evidence suggests that the experience of stress can be communicated between individuals via chemosensory cues. It was proposed that upon activation of the sympathetic-adrenal medullary (SAM) system, operating closely with the hypothalamus-pituitary-adrenal (HPA) axis, individuals release sweat, which includes physiological markers of anxiety/fear (de Groot et al., 2015). In response to threat, ranging from situations inducing acute fear (such as skydiving, e.g., Mujica-Parodi et al., 2009) to situations inducing acute anxiety (such as anticipation of an oral examination, e.g., Prehn et al., 2006; Prehn-Kristensen et al., 2009), the release of “alarm" signals induces a partial fear state in those exposed to the chemosensory compounds (Prehn et al., 2006; Mujica-Parodi et al., 2009; de Groot et al., 2012). This includes fear-related behavioral and physiological outcomes such as improved cognitive performance (Chen et al., 2006), a bias toward identification of faces as more fearful (e.g., Zhou and Chen, 2009), increased visual field size, and enhanced sensory intake (de Groot et al., 2012), to name just a few. The successful transmission of the “alarm” signals is believed to serve the adaptive function of enhancing sensory vigilance, preparing the organism for environmental dangers (for a review see Stevenson, 2010).

Although current support for the role of chemosensory “alarm” signals in modulating specific emotional, cognitive and physiological processes is abundant (Stevenson, 2010; Pause, 2012), little is known about the impact of these signals on neurophysiological responses during an *actual* threatening context. Given that in everyday life, exposure to chemosignals rarely occurs without a relevant contextual background, it seems critical to assess the impact of chemosensory “alarm” signals during an actual threat. Thus, the current study investigated the impact of chemosensory cues of anxiety on one of the most distressing social situations, i.e., social exclusion. Social exclusion is considered a social danger, as it threatens the basic human need to belong, which is necessary for survival and well-being (Bowlby, 1969; Baumeister and Leary, 1995).

To examine whether chemosensory cues signaling anxiety modulate neuronal effects to social exclusion we first collected axillary sweat from donors in anticipation of an oral examination at a university, which has been linked to experiences of anxiety and the release of emotional chemosignals (e.g., Prehn-Kristensen et al., 2009). We then employed a widely used and well-validated paradigm to study social ostracism in the laboratory environment – Cyberball (Williams et al., 2000; Williams and Jarvis, 2006). In this task participants play a ball-tossing game with two individuals (in reality simulated by the computer) in which, after a series of inclusion trials, they are eventually excluded from the game. The exclusion from Cyberball has been shown to pose a threat to the basic human needs of belonging, feeling in control, maintaining self-esteem, and experiencing a meaningful existence (Williams, 2007; Eisenberger, 2012).

The central question of the current research was whether the communication of anxiety via chemosensory signals modulates the neuronal responses to social exclusion. The evidence suggesting that chemosensory “alarm” cues enhance salience of fear-related socio-emotional cues (Zhou and Chen, 2009) argues for augmented experience of social rejection following the exposure to the anxiety chemosignals. Similarly, studies pointing to emotion contagion via chemosensory “alarm signals” (Prehn et al., 2006; Mujica-Parodi et al., 2009; de Groot et al., 2012) suggest enhanced contagion of anxiety in the context of a distressing situation. Further, the results showing increased fear contagion when olfactory fear is paired with another modality (de Groot et al., 2014) also support this hypothesis. Cumulatively, the expected increased negative experience of social exclusion could be a collective result of chemosensory anxiety and a distressing context. Alternatively, given that chemosensory anxiety cues are potential social threat signals, it cannot be precluded that they are associated with enhancing reappraisal of social rejection to promote fitness. Specifically, from an evolutionary perspective, the successful chemosensory communication of another person’s anxiety could be expected to lead to dissociation from the negative experience of social exclusion, in order to enhance productive coping mechanisms during the potentially harmful situation. This hypothesis is supported by an abundance of animal and human studies suggesting a key role for chemosensory “alarm” cues in preparing the organism to tackle a hazardous situation via boosting physiological arousal (Prehn et al., 2006; Inagaki et al., 2008; Pause et al., 2009) and enhancing sensory vigilance (Brown et al., 2004; Chen et al., 2006; Zhou and Chen, 2009; de Groot et al., 2012). These processes may be initiated in preparation to withdraw from the threatening situation (for a review see Pause, 2012).

Taken together, the current study assessed the neuronal implications of exposure to chemosensory anxiety cues in a threatening context of social exclusion. Does the smell of another’s person anxiety make us more vulnerable to social exclusion or more prepared to cope with the difficult situation? Increased activity in regions typically involved in processing of social exclusion, including anterior cingulate cortex, medial orbitofrontal cortex, insula, and in regions previously reported to play a role in the processing of socio-emotional information, including the amygdala, hippocampus, and superior temporal gyrus, would support the first answer. By contrast, diminished activity in the regions typically implied in processing of social exclusion would argue for down-regulation of negative feelings associated with ostracism.

## Materials and Methods

### Ethics Statement

The local ethics committee at the Medical Faculty of RWTH Aachen University approved the current study. The experimental protocol was carried out in accordance with the provisions of the World Medical Association Declaration of Helsinki. All participants gave written informed consent and were reimbursed for their time.

### Participants

All volunteers were part of a larger study on the effects of anxiety chemosignals on social cognition. Ten healthy males were recruited as sweat donors. Twenty-four healthy participants (14 men and 10 women) were recruited to take part in the Cyberball study, as sweat recipients. Only males were chosen to donate their sweat, as the apocrine glands in the male underarm area are known to be larger (Doty, 1981). Both genders were included in the fMRI study, as previous research indicated that male stress sweat induces similar neural responses in both gender recipients (Radulescu and Mujica-Parodi, 2013). All Cyberball participants (18–29 years, *M* = 24.33 years, *SD* = 2.91) were screened for fMRI contraindications. In addition, we included only right-handed (Edinburgh Inventory; Oldfield, 1971), non-smoking individuals, who did not suffer from any neurological nor psychiatric illnesses (as assessed via Structured Clinical Interview for DSM-IV, SCID, First et al., 1995), nor showed signs of depression (Beck Depression Inventory; Beck et al., 1961; *M* = 2.9; *SD* = 3.9). Participants’ olfactory function, specifically odor identification, was assessed with Monex 40 (Freiherr et al., 2012). According to this task, all participants were normosmic (range: 27–36, *M* = 31.54, *SD* = 2.90).

### Materials

#### Chemosensory Stimuli

The olfactory stimuli consisted of sweat samples collected from males undergoing an important oral examination (anxiety condition) and exercising at a stationery bicycle (sports condition). The sweat donors were invited to take part in the study if they anticipated an important oral examination about which they felt nervous. In addition, their participation was only possible if they reported to be: of Caucasian origin, heterosexual, non-smokers, aged between 18 and 40 years-old, healthy, physically fit and not taking any medication. Ten males (22–33 years, *M* = 26.40 years; *SD* = 3.75) who fulfilled these criteria were recruited to donate their sweat. They were asked to follow several rules starting 2 days prior to the sweat donation (consistently with, e.g., Zhou and Chen, 2009; de Groot et al., 2012). These included not going into the swimming pool or sauna, not consuming garlic, onion, asparagus, curry, and strongly spiced meals, not drinking alcohol and coffee, sleeping alone, not using deodorant, after-shave, scented creams, and perfumes. In addition, they were asked to use scent-less shampoo and soap provided to them by the experimenter starting 2 days prior to the donation as well as to wash their sheets with an odor-less detergent. Before the sweat donation session, they were asked to shower and wear clothes, which they had washed with an odor-less detergent provided by the experimenter. All the participants reported following these rules.

The experimental protocol for sweat collection was based on Prehn-Kristensen et al.’s (2009) design. Specifically, the olfactory stimuli were gathered from donors’ underarm area with cotton pads attached with plasters for sensitive skin. In the anxiety condition, participants’ sweat was collected during anticipation of an important oral examination for 60 min. In addition, at that time, participants’ salivary samples were gathered to assess cortisol levels: 60 min before the examination (t0), 30 min before the examination (t1), right before the examination (t2), and right after the examination (t3) using salivettes (Sarstedt, Nuembrecht, Germany). Participants were further asked to complete a Self-Assessment Manikin (Bradley and Lang, 1994) and to evaluate the intensity of six basic emotions (anxiety, joy, surprise, anger, sadness, disgust; Ekman and Friesen, 1975) on visual analog scales, 5 min before the examination. Shortly before the examination the sweat pads were removed. The sports condition consisted of a 10-min introduction to the procedure, three sets of cycling on a stationary bike, 10 min in duration each, with 10 min breaks in between the sets. The salivary samples were collected right at the beginning of the session (t0) as well as 30 (t1), 60 (t2), and 90 min after the start (t3). The Self-Assessment Manikin and Basic Emotions questionnaire were administered after the last bicycle set. Shortly after that the sweat pads were removed. Upon completion of the sweat collection procedure the pads from both sessions were cut into 8 pieces each, blinded by an experimenter not directly involved in this research, sealed in odor-free freezer bags separately per condition and stored at -80° Celsius until the day of the examination. The four pieces of the pads per condition, originating from 4 different donors, were defrosted 30 min before the experiment and placed in odorless teabags. During the fMRI experiment, the olfactory stimuli were attached under participants’ noses with an odorless strap for the duration of the experimental trial (see *Procedure* for more details). Care was taken to ensure that the chemosensory stimuli did not come into direct contact with participants’ skin. Each chemosensory stimulus was prepared and used for one participant only.

#### Cyberball Task

**FIGURE 1:**
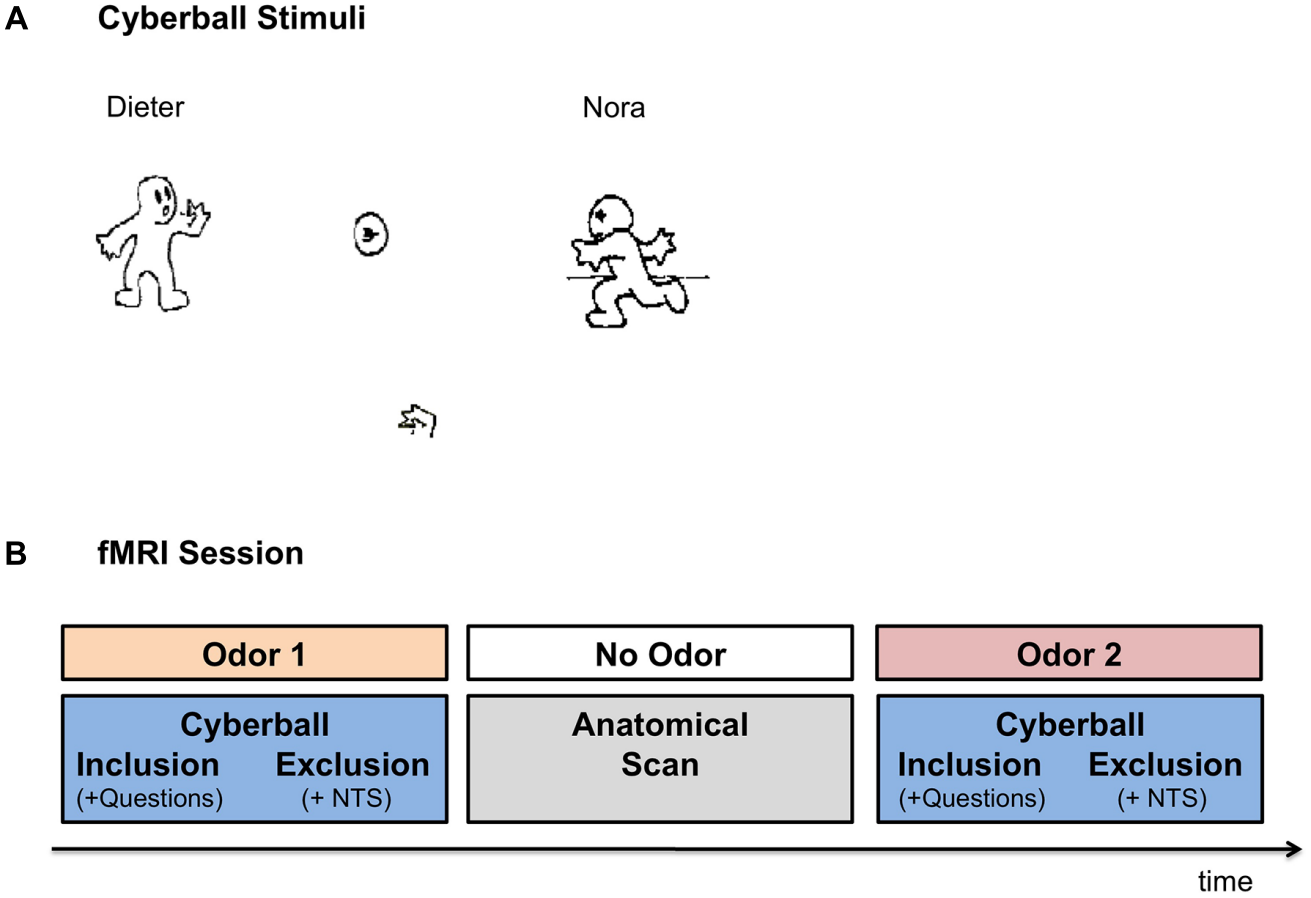
**Experimental Setup. (A)** Cyberball game – the visual interface. Participants were presented with the icons via MRI compatible goggles. The participants were represented by the hand icon, at the bottom of the screen. Once the participant received the ball, they were allowed to toss it to one of the other two players (in reality simulated by computer). In the inclusion condition participants were part of the game throughout the trial. In the exclusion condition, the participants received the ball in the first 10 throws, after which they were excluded from the game for the rest of the trial (∼50–70 s). **(B)** Schematic Illustration of the FMRI session. In the fMRI session participants completed two runs of the Cyberball game under exposure to anxiety and sports chemosensory cues, presented in a counterbalanced order. In each Cyberball run the inclusion condition preceded the exclusion condition. Following the inclusion condition, participants were asked how angry they felt, how happy they felt, how much they liked Dieter and how much they liked Nora (i.e., Questions). Following the exclusion condition, participants completed the Need Threat Scale (NTS). In between the two runs of the Cyberball game the anatomical scan was carried out for approximately 10 min, in which no chemosensory cues were presented.

Participants completed the Cyberball task (Williams et al., 2000), a virtual ball-tossing game developed to study social ostracism. The game consists of cartoon images representing other players in the upper corners of the screen and a hand representing the participant at the bottom of the screen, tossing the ball among each other (see **Figure 1A** for illustration). In the current version, the names of the players –“Dieter” and “Nora” – were displayed on the screen next to the animated cartoons. The participants were informed that they were connected to the other two individuals over the Internet, while in reality the game was simulated by the pre-set computer program. The game included two conditions: social inclusion and social exclusion. The participants completed two rounds of the game, one each under exposure to anxiety vs. control sweat cues, presented in a counterbalanced order (see **Figure 1B** for a schematic illustration of the session). For each odor, an inclusion condition was followed by an exclusion condition. Each condition started with a display indicating that the computer was connecting to the other players. The fixation cross was then presented for 15 s, after which the trial began. The participants could toss the ball to one of the two players, by pressing “left” with their index finger or “right” with their middle finger, on a response box. Each trial was set for 60 throws, with the opponents tossing the ball after 0.5–3.0 s of waiting time (determined randomly). In the inclusion condition, participants played with the two opponents throughout the trial. Each player received the ball on approximately 50% of the throws. In the exclusion condition, the participants received the ball for the first 10 throws, after which they were excluded from the game and did not receive the ball for the rest of the trial (∼50–70 s) while other players tossed the ball among themselves. Following the inclusion condition participants were asked how happy and how angry they were as well as how much they liked the female player and the male player. Following the exclusion condition, participants completed the Need-Threat scale (Williams et al., 2000, 2002) assessing participants’ four fundamental needs: belonging (e.g., I felt I belonged to the group), self-esteem (e.g., I felt good about myself), meaningful existence (e.g., I felt non-existent), and control (e.g., I felt powerful). In order to maintain the cover story, the Need-Threat scale was administered to the participants only after the exclusion condition.

### Procedure

Before and after the fMRI session participants were asked to evaluate the olfactory stimuli with respect to their valence, intensity and pleasantness. During fMRI scanning, the olfactory stimuli were attached under participants’ noses for the duration of the Cyberball run (inclusion condition followed by an exclusion condition plus the accompanying questionnaires). The participants completed two rounds of the game under exposure to the anxiety vs. control sweat cues. The order of olfactory stimuli presentation was counterbalanced across the participants and both the participants and the experimenters were blinded to the nature of the olfactory cues. For a break between the two chemosensory stimulations, the two Cyberball trials were separated by a 10-min anatomical scan, during which no olfactory stimuli were presented. See **Figure 1B** for a schematic illustration of the fMRI session.

### fMRI Data Acquisition

Functional MRI data were acquired in a three Tesla Tim Trio MR Scanner (Siemens, Erlangen, Germany) at the Department of Psychiatry, Psychotherapy, and Psychosomatics at the Hospital of the RWTH Aachen University. Functional images were collected with an echo-planar imaging (EPI) T2^∗^-weighted contrast sequence sensitive to blood oxygenation level dependent (BOLD) changes (echo time [TE] = 28 ms, repetition time [TR] = 2 s, flip angle [α]: 77°, voxel size: 3 mm × 3 mm × 3 mm, 64 × 64 matrix, field of view [FOV]: 192 mm × 192 mm, slice thickness: 3.0 mm, gap: 0.75 mm, number of slices: 34 axial slices, whole-brain, slice acquisition sequence: ascending, 790 volumes per run).

### Behavioral Data Analyses

The behavioral data were analyzed using IBM SPSS Statistics 20 (SPSS IC, Chicago, IL, USA). The effect sizes are reported according to Cohen’s *d* (1988) for paired-sample comparisons and all *post hoc t*-tests.

#### Sweat Donor Physiological and Questionnaire Data

The cortisol levels (in μg/dl) of the sweat donors were extracted at the diagnostic laboratory of the Medical Department of RWTH Aachen University from salivary samples (LDZ: Labordiagnostisches Zentrum Aachen). Three sweat donors were found to produce insufficient salivary amount necessary for the extraction of cortisol values at one of the collection points. The cortisol values of the remaining subjects (*n* = 7) were compared in a 2 (anxiety smell, sports smell) × 4 (time 0, time 1, time 2, time 3) repeated measures analysis of variance (rmANOVA). Follow up comparisons were carried out for each of the salivary sampling points (including the available salivary data for each time point) using Wilcoxon signed-rank test. In addition, the donors’ emotional experiences (Basic Emotions and SAM emotions) in the anxiety and sports conditions were compared using Wilcoxon signed-rank test. The Wilcoxon signed-rank test was chosen for these data due to the small sample size of sweat donors (*n* = 10).

#### Odor Differentiation

In order to evaluate whether participants could differentiate the smells with respect to their pleasantness, intensity and valence, the participants’ (*n* = 21) ratings of sweat stimuli against these criteria were compared in separate 2 (anxiety smell, sports smell) × 2 (time 1, time 2) × 2 (men, women) rmANOVAs. Three ratings of the participants were not recorded due to measurement errors.

#### Cyberball Behavioral Data

In the Cyberball game, participants’ contentment was compared in the exclusion condition versus the inclusion condition (manipulation check) with a paired samples *t*-test, across chemosensory conditions. In addition, participants’ evaluation of how much they liked the male and the female opponent in the game, how happy and how angry they felt following the inclusion trial were analyzed with separate 2 (anxiety smell, sports smell) × 2 (men, women) rmANOVAs. Similarly the ratings of the experience of fundamental needs of belonging, self-esteem, control and meaningful existence (NTS; Williams et al., 2000, 2002) following exclusion were analyzed with 2 (anxiety smell, sports smell) × 2 (men, women) rmANOVAs.

### fMRI Data Analyses

The neuroimaging data were preprocessed and analyzed using SPM8 (Wellcome Trust Center for Neuroimaging^1^) implemented via Matlab 7.10 (MathWorks). Data from two participants who exhibited excessive motion (more than 3 mm in any direction) were excluded from the analyses: the final participant sample for the analyses consisted of 22 participants (12 men and 10 women).

#### Preprocessing

The fMRI data were preprocessed according to standard preprocessing steps (including realignment, coregistration, normalization, and smoothing). The functional scans were first realigned using a two-pass procedure. In this procedure, the first pass—the first scan, and the second pass—the mean scan, were substituted as reference image. Subsequently, the anatomical scans were coregistered to the mean EPI scan. The coregistered images were used for the estimation of spatial normalization parameters using unified segmentation approach (Ashburner and Friston, 2005). The normalization parameters applied to the images transformed them into the standard space as defined by the Montreal Neurological Institute (MNI) and resampled the images to a voxel size of 2 mm × 2 mm × 2 mm. Lastly, smoothing of the images was conducted with a Gaussian kernel of 8mm full-width-at-half-maximum.

#### First Level Analyses

In the first level analyses, the onset and duration vectors for separate Cyberball conditions (i.e., exclusion and inclusion blocks) under the two chemosensory conditions (i.e., anxiety and sports) were convolved with hemodynamic response function (HRF). In addition, the onsets and duration vectors for the sources of noise (i.e., instructions, questionnaires, and waiting time) were modeled out in a separate regressor of no interest. The mean across time for each voxel was modeled by a constant term and low-frequency drifts were removed using a high-pass filter with a cutoff period of 512 s. Temporal correlations were modeled by a first-order regression process as implemented in SPM.

#### Second Level Analyses

Second level analyses were conducted using GLM Flex, (extension to SPM8, see GLM Flex^2^) in which the experimental plan included the following factors: smell (anxiety, sports) and ostracism condition (inclusion, exclusion).

#### Whole Brain Analyses

Whole brain analyses targeted at examining the impact of anxiety chemosensory cues on the experience of social ostracism. We contrasted neural activity during exclusion relative to inclusion separately for: (a) the exposure to chemosensory control cues, (b) chemosensory anxiety cues, and (c) chemosensory anxiety cues – chemosensory control cues. Moreover, we conducted a whole brain smell (anxiety, sports) × condition (inclusion, exclusion) interaction. xJView^3^, a viewing program for SPM, was used for exploring and processing of the contrasts and MarsBaR toolbox for SPM^4^ was used for exploring and processing of the interaction. Additionally, the Anatomy toolbox for SPM (Eickhoff et al., 2005) and the xjView were used for anatomical localization.

#### Volume of Interest Analyses

In order to clarify which condition in our 2 (anxiety smell, sports smell) × 2 (inclusion, exclusion) design, drove the interaction, we identified activation clusters volumes of interest (VOIs) within socio-emotional regions, using MarsBaR toolbox from all the significantly activated brain regions in the whole brain interaction. The VOIs included the areas previously implied in the Cyberball paradigm (i.e., orbitofrontal cortex and anterior cingulate) as well as the areas involved in social cognition and memory (i.e., superior temporal gyrus, hippocampus, inferior frontal gyrus). Although we are well-aware of the broad involvement of these areas in a wide range of functions, we chose the specified regions based on research (see below), suggesting an intimate relationship with measured processes, of which activation/deactivation patterns in the context of chemosensory anxiety could point to mechanisms by which chemosensory anxiety influences the experience of social exclusion. The VOIs included:

(1) Orbitofrontal cortex and anterior cingulate (associated with rumination and persisting negative affect, Kohn et al., 2013; as well as negative experience of social exclusion, Eisenberger, 2012);
(2) Right middle temporal gyrus and right superior temporal gyrus (linked to perception of familiar places and scenes, Tempini et al., 1998; Leveroni et al., 2000, and social cognition and theory of mind, Carrington and Bailey, 2009);
(3) Right hippocampus (involved in memory processes, Brewer et al., 1998; Wagner et al., 1998; Morris, 2007);
(4) Left inferior frontal gyrus (involved in response inhibition, emotion regulation, as well as processing of salience; Hampshire et al., 2010; Kohn et al., 2014).

For information about volume, center of mass, peak MNI coordinates, cluster size, and the peak intensity (*T* statistic), refer to **Table 3**.

The mean beta estimates (approximating the activation strength) values in the four clusters were extracted for each subject and each condition against the implicit baseline, and subjected to separate 2 × 2 × 2 rmANOVAs comprising factors: smell (anxiety, sports) × ostracism condition (exclusion, inclusion) × gender (men, women). *Post hoc* analyses were calculated using paired-samples *t*-tests. The comparisons of interest included: anxiety exclusion – anxiety inclusion, sports exclusion – sports inclusion and anxiety exclusion – sports exclusion.

#### Correction for Multiple Comparisons

To correct for multiple comparisons, we applied extent threshold correction as defined by Monte Carlo simulations (3DClustSim; implemented in AFNI; Cox, 1996). This procedure prevents false discoveries resulting from multiple testing. For a threshold at the voxel level of *p* = 0.001 uncorrected, and spatial properties of the current study, 10,000 simulations resulted in an extent threshold of 72 resampled voxels.

## Results

### Sweat Donors

#### Cortisol

Sweat donors showed higher cortisol levels when awaiting an oral examination than during ergometer training as revealed by a main effect of condition; *F* = 8.774, *p* = 0.025. The Wilcoxon Signed-rank test indicated that there was a significant difference in the cortisol values overall (average rank of 5.50 in the anxiety condition vs. average rank of 0.00 in the sports condition, *Z* = -2.366, *p* = 0.018), as well as specifically at time 0 (average rank of 5.50 vs. 1.00, *Z* = -2.547, *p* = 0.011), time 1 (average rank of 5.50 vs. 0.00, *Z* = -2.805, *p* = 0.005), time 2 (average rank of 4.50 vs. 0.00, *Z* = -2.521, *p* = 0.012), and time 3 (average rank of 6.00 vs. 1.50, *Z* = -2.310, *p* = 0.021). No other main effects nor interactions were observed (all *p* > 0.05). See **Table 1** for an overview of participants’ cortisol values.

**Table 1:** Descriptive statistics for sweat donors’ cortisol levels in the anxiety and sports conditions at times 0, 1, 2, 3, and overall [Mean (SD) and Median].

Condition	Time 0		Time 1		Time 2		Time 3		Overall	
	*M (SD)*	*Mdn*	*M (SD)*	*Mdn*	*M (SD)*	*Mdn*	*M (SD)*	*Mdn*	*M (SD)*	*Mdn*
Anxiety	0.68 (0.34)	0.61	0.59 (0.30)	0.60	0.54 (0.28)	0.41	0.65 (0.36)	0.65	0.62 (0.27)	0.61
Sports	0.46 (0.20)	0.50	0.36 (0.14)	0.33	0.34 (0.14)	0.32	0.34 (0.10)	0.32	0.37 (0.14)	0.39

#### Emotions

**FIGURE 2:**
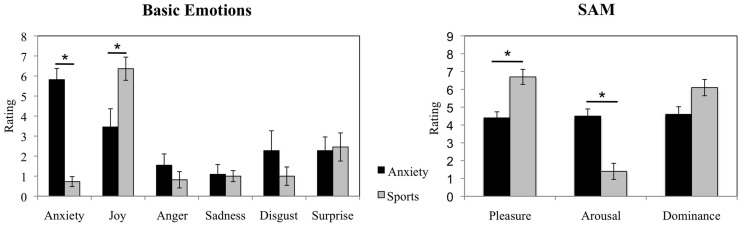
**Sweat donors’ emotional responses in the anxiety vs. sports conditions**. Sweat donors’ evaluations of basic emotions and ratings in Self-Assessment Manikin during anticipation of an oral examination and during ergometer training. The figure depicts participants’ mean ratings and standard errors. ^∗^*p* < 0.05.

The Wilcoxon Signed-rank test revealed that the donors in the anxiety condition showed an increase in reported anxiety as compared to the sports condition (average rank of 5.50 vs. 0.00, *Z* = -2.814, *p* = 0.005). In addition, it revealed a decrease in experienced joy before the examination than during the ergometer training (average rank of 2.50 vs. 6.79, *Z* = -2.053, *p* = 0.040, see **Figure 2** for a visual depiction of mean rating differences). No significant differences in the experience of other basic emotions were observed (all *p* > 0.05). In addition, in the Self-Assessment Manikin, the donors reported feeling less pleasure while awaiting an examination (average rank of 0.00 vs. 5.50, *Z* = -2.680, *p* = 0.007) and more arousal while anticipating an examination as compared to the training (average rank of 5.50 vs. to 0.00, *Z* = -2.831, *p* = 0.005, see **Figure 2** for visual depiction of mean rating differences). The donors did not report differences in experienced dominance (*p* > 0.05).

### Olfactory Samples

**FIGURE 3:**
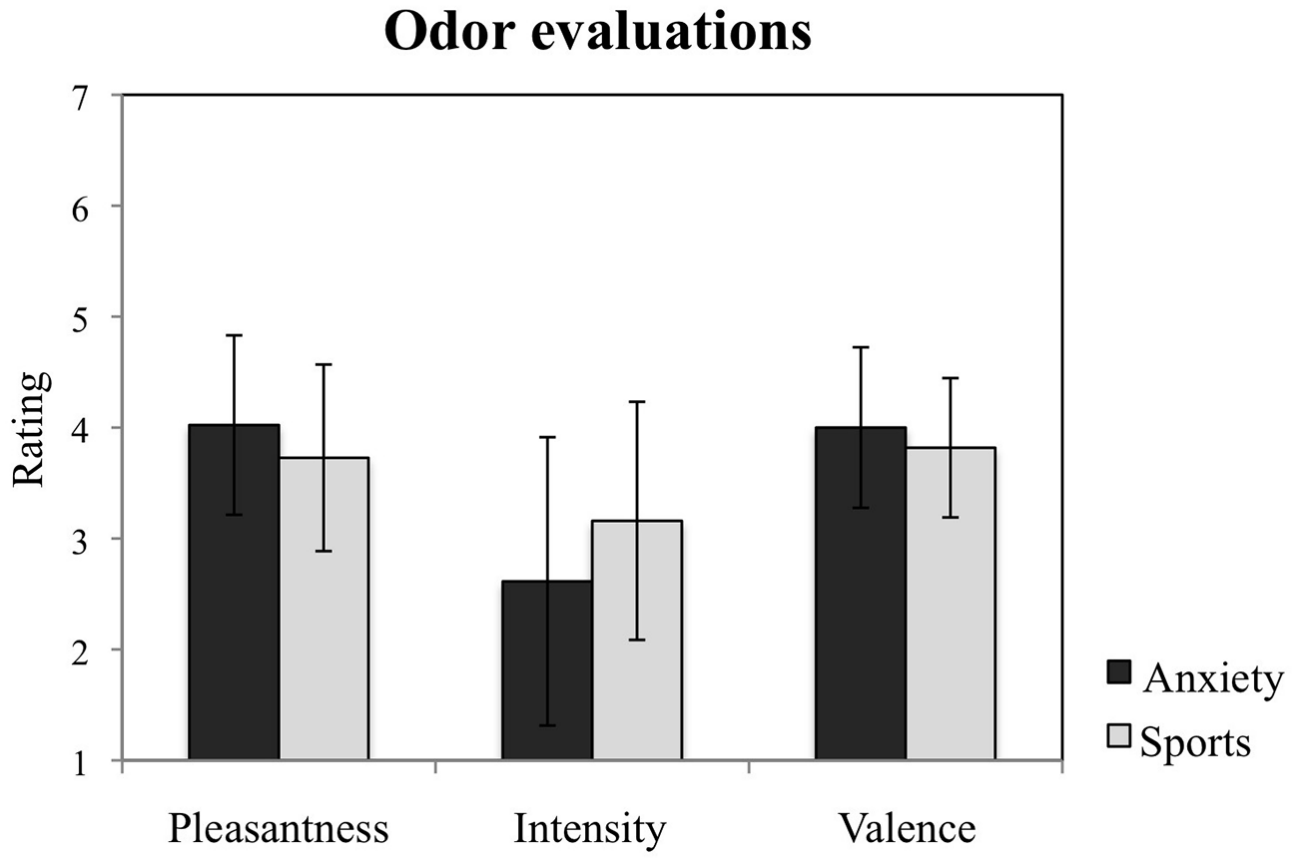
**Study participants’ evaluations of the chemosensory cues**. Participants’ ratings of the odors from the anxiety and the sports conditions with regard to their pleasantness, intensity, and valence. The figure depicts participants’ mean ratings and standard errors.

The participants exposed to the olfactory samples collected from the two situations reported no difference in odor characteristics with regard to pleasantness, *F* = 3.775, *p* = 0.067; intensity, *F* = 2.234, *p* = 0.151, and valence, *F* = 1.055, *p* = 0.317 (see **Figure 3**). No other main effects nor interactions with time nor gender for any of these measures were observed (all *p* > 0.05).

### Cyberball

#### Manipulation Check

**FIGURE 4:**
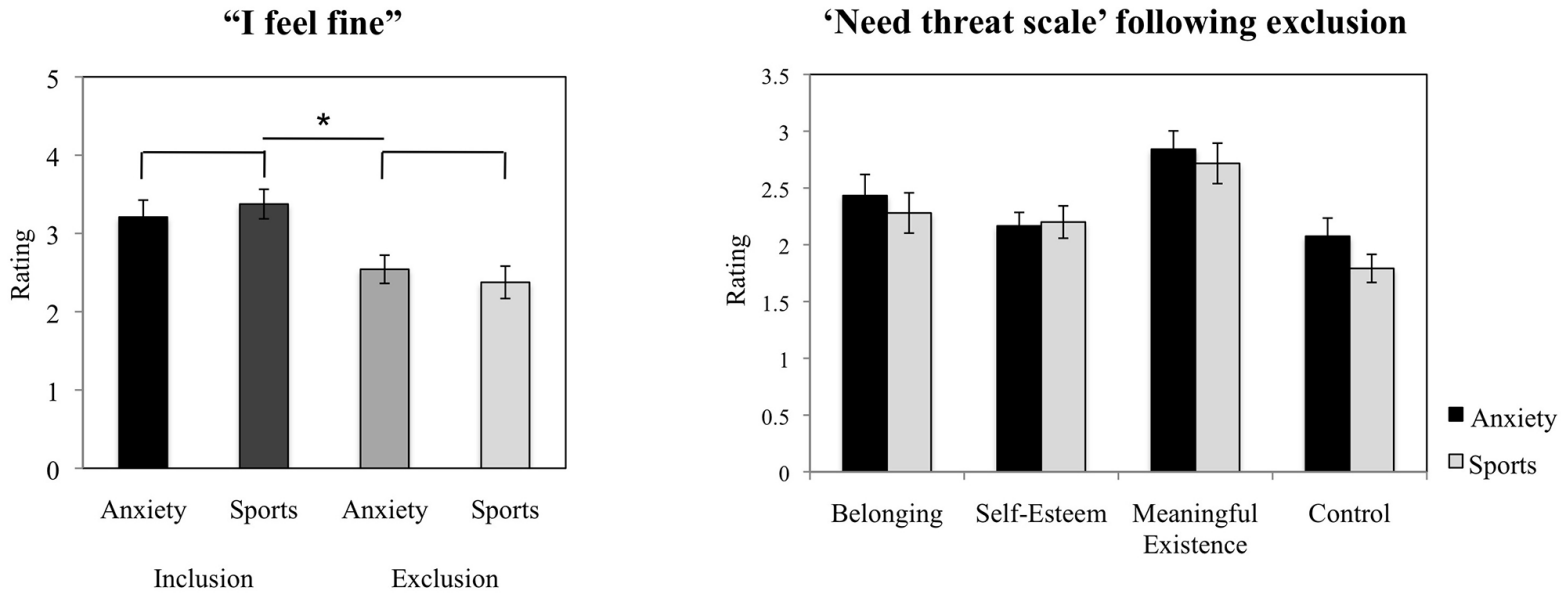
**Cyberball task**. Participants’ ratings of contentment in the exclusion condition compared to the inclusion condition (manipulation check), and evaluation of the experience of basic needs (NTS) following social exclusion. The figure depicts participants’ mean ratings and standard errors. ^∗^*p* < 0.05.

Across chemosensory conditions, the participants reported feeling significantly less contented in the exclusion condition (*M* = 2.46, *SD* = 0.86) as compared to the inclusion condition [*M* = 3.29, *SD* = 0.79; *t*(23) = 3.815, *p* = 0.001, *d* = 1.01, see **Figure 4**].

#### The Experience of Inclusion Under Chemosensory Cues

Following inclusion, under anxiety sweat as compared to sports sweat the participants did not report differences in the experience of happiness (*F* = 0.750, *p* = 0.396), nor anger (*F* = 3.305, *p* = 0.083), nor in how much they liked the male (*F* = 0.013, *p* = 0.909), nor the female participant (*F* = 0.118, *p* = 0.735). Further, the scores were not influenced by gender (all *p* > 0.05).

#### The Experience of Exclusion Under Chemosensory Cues – Need Threat Scale

The chemosensory cues of anxiety did not exert effects on the feeling of belonging (*F* = 1.010, *p* = 0.326), self-esteem (*F* = 0.376, *p* = 0.546), the experience of feeling “in control” (*F* = 3.399, *p* = 0.079), nor meaningful existence (*F* = 0.583, *p* = 0.453, see **Figure 4**). Further, the scores were not influenced by gender (all *p* > 0.05).

## fMRI

### Neural Responses to Ostracism

#### Whole Brain Analyses

To assess the effect of chemosensory exposure on neural responses to social exclusion, we examined neural regions that differed in response to social exclusion (compared to inclusion) when participants were exposed to: (a) chemosensory sports (control) cues, (b) chemosensory anxiety cues, (c) chemosensory anxiety cues – chemosensory sports cues. Further, we conducted a smell (anxiety, sports) × condition (exclusion, inclusion) interaction.

##### Chemosensory sports (control) cues

**FIGURE 5:**
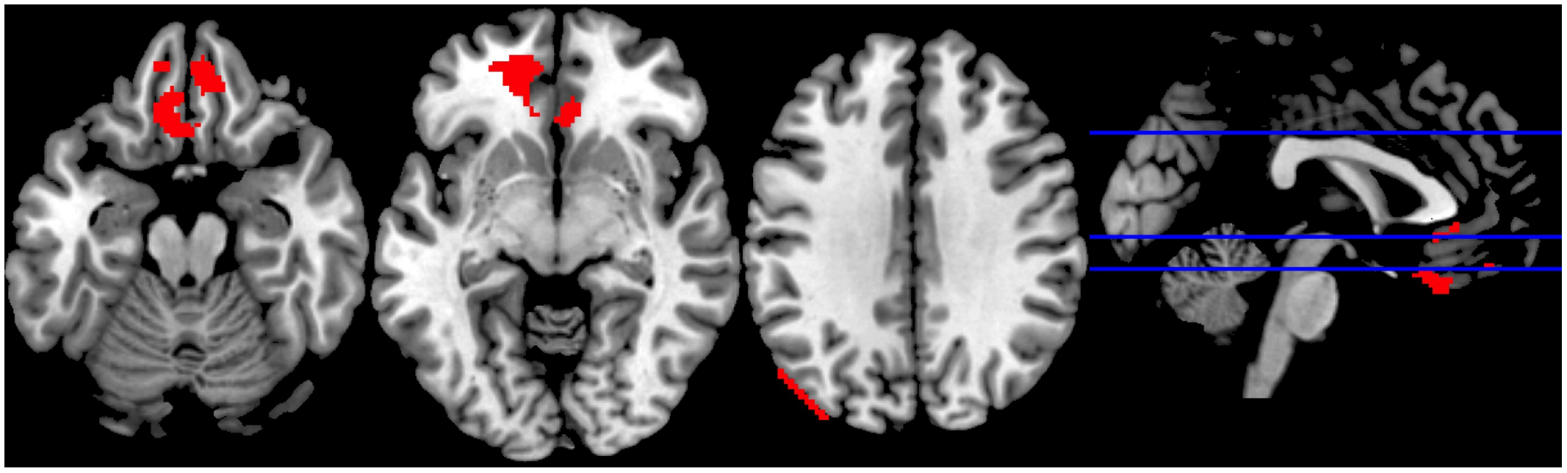
**Sports Exclusion > Sports Inclusion (Whole Brain analyses)**. Significant clusters at *p* < 0.001, with extent threshold = 72 voxels (corresponding to Monte Carlo correction) in the contrast Exclusion > Inclusion under the exposure to chemosensory sports (control) cues.

We observed increased activity in the clusters encompassing: (1) rectal gyrus, superior orbital gyrus, anterior cingulate, and medial frontal gyrus; (2) anterior cingulate, rectal gyrus, and medial frontal gyrus, (3) superior occipital gyrus, angular gyrus and middle temporal gyrus (for details see **Table 2**; **Figure 5**).

**Table 2:** Neural activations in the contrast Exclusion > Inclusion for: (a) sports chemosensory condition; (b) anxiety chemosensory condition; (c) anxiety chemosensory condition – sports chemosensory condition.

Contrast	Brain regions	Hemisphere	Peak MNI coordinates	*k*	Peak intensity
**Control “sports” cues** Exclusion > Inclusion	Rectal gyrus, superior orbital gyrus, anterior cingulate, medial frontal gyrus	L	-16, 46, -8	492	7.388
	Anterior cingulate, rectal gyrus, medial frontal gyrus	R, L	6, 34, -2	246	5.864
	Angular gyrus, superior occipital gyrus, middle temporal gyrus	L	-46, -82, 28	87	7.832
**Anxiety cues** Exclusion > Inclusion	No suprathreshold activation				
**Anxiety – control** Exclusion > Inclusion	No suprathreshold activation				

##### Chemosensory anxiety cues

No significant suprathreshold activations were observed in the exclusion condition (as compared to inclusion condition).

##### Chemosensory anxiety cues – chemosensory sports cues

No significant suprathreshold activations were observed in the exclusion condition (as compared to inclusion condition).

##### Smell (anxiety, sports) × condition (exclusion, inclusion) interaction

Significant activations were observed in 11 clusters. For information about regions within the cluster, hemisphere, volume, center of mass, peak MNI coordinates, cluster size, and peak intensities of the volumes, please see **Table 3** and Supplementary Material for the figure depicting the interaction.

**Table 3:** Information regarding brain regions, hemisphere, volume, center of mass, peak MNI coordinates, cluster size, and peak intensity (*T* statistic) for significantly activated clusters in the whole brain smell × condition interaction.

Cluster(regions)	Hemisphere	Volume (mm)	Center of mass	Peak MNI coordinates (X, Y, Z)	*k*	Peak intensity
**^∗^Orbitofrontal cortex, anterior cingulate, medial frontal gyrus**	L, R	5216.0	-3.9, 43.0, -14.9	-12, 44, -14	652	-7.2228
**^∗^Middle temporal gyrus, superior temporal gyrus**	R	1384.0	63.3, -38.5, -0.3	62, -40, 2	173	-5.5097
**^∗^Hippocampus**	R	1336.0	28.7, -36.4, -9.8	32, -38, -10	167	-6.6943
**^∗^Inferior frontal gyrus**	L	848.0	-45.8, 23.6, 15.7	-46, 26, 18	106	-5.79
Cerebellum, lingual gyrus, fusiform gyrus	L, R	16776.0	8.67, -52.4, -19.3	18, -52, -4	2097	-7.2425
Brainstem, midbrain, thalamus	L, R	4696.0	0.927, -20.6, -11.6	4, -24, -22	587	-7.2277
Transverse temporal gyrus, thalamus, superior temporal gyrus, insula, putamen	L	2320.0	-28.4, -22.7, 9.59	-34, -28, 10	290	-6.598
Caudate, putamen, thalamus	R	2128.0	21.2, -7.83, 11.7	22, -8, 20	266	-5.0169
Cerebellum	R	1456.0	47.4, -56.9, -38.7	52, -56, -40	182	-7.0123
Middle temporal gyrus, Broadmann area 21	R	992.0	50.3, -6.82, -19.4	58, 0, -26	124	-4.5159
Transverse temporal gyrus, superior temporal gyrus	R	800.0	40.8, 8.4, 7.3	44, -26, 4	100	-5.6465

*The highlighted regions constitute the Volumes of Interest (VOIs) for which beta estimates were extracted for analyses aimed at disentangling the interaction*.

#### VOI Analyses

Four VOIs were selected due to their contextual importance to underlying processes (see methods section for further information) in order to disentangle the smell × ostracism interaction.

##### Orbitofrontal cortex/anterior cingulate

**FIGURE 6:**
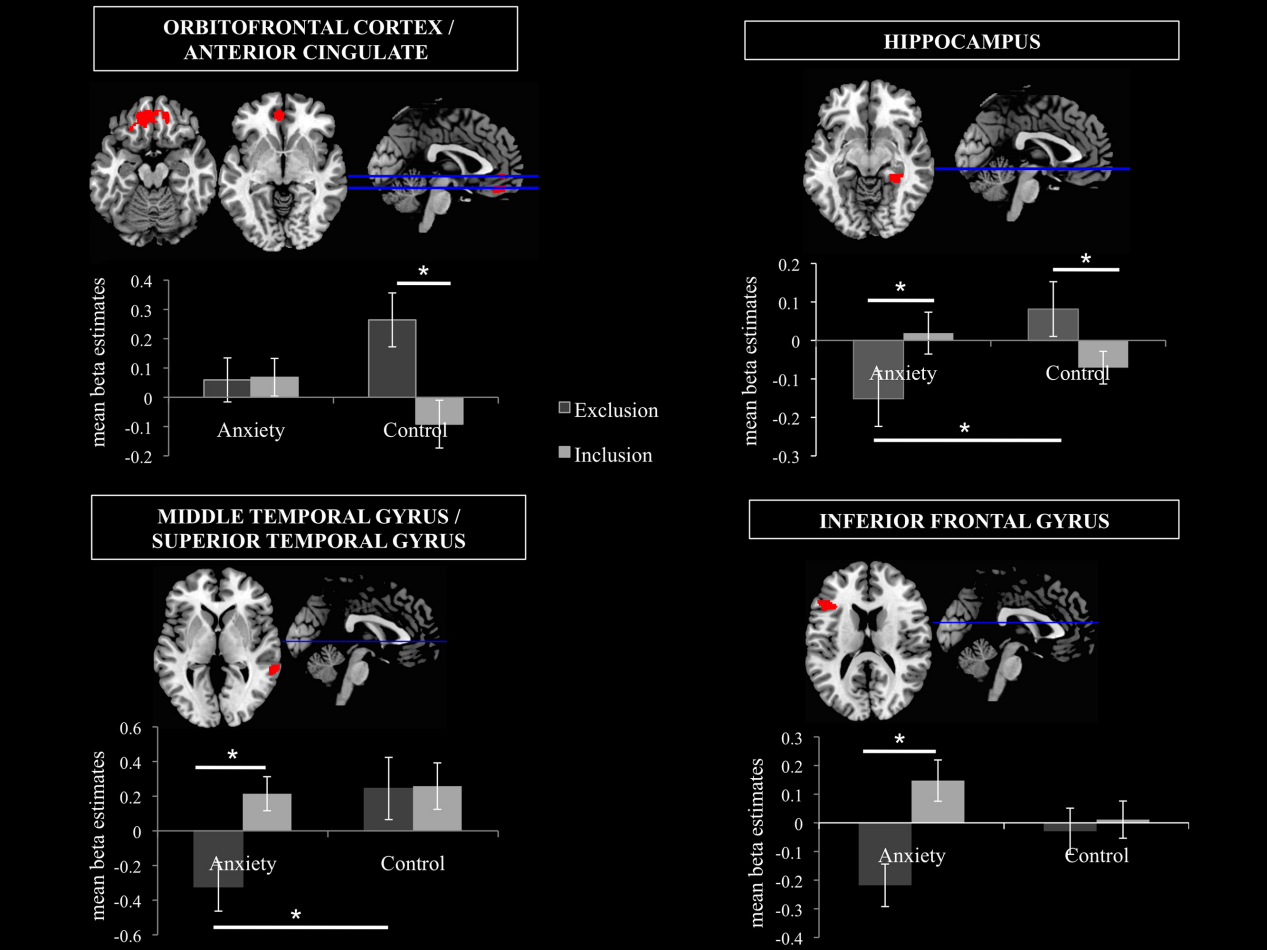
**Volumes of Interest (VOIs)**. Mean beta estimates *post hoc* comparisons in the 4 VOIs are displayed in order to disentangle the smell × ostracism condition interaction. The significant clusters are at *p* < 0.001, with extent threshold = 72 voxels (corresponding to Monte Carlo correction). ^∗^*p* < 0.05.

A significant main effect of ostracism condition (*F* = 14.913, *p* = 0.001) was identified in the VOI encompassing the superior orbitofrontal cortex and the anterior cingulate (Peak MNI coordinate region: -12, 44, -14, see **Figure 6**), with participants showing increased activity in this region in the exclusion condition (*M* = 0.16, *SD* = 0.25) and relative inhibition in the inclusion condition [*M* = -0.01, *SD* = 0.24, *t*(21) = -3.990, *p* = 0.001, *d* = 0.69]. In addition, a significant smell × condition interaction (*F* = 10.664, *p* = 0.004) revealed that under the chemosensory control cues, the participants showed higher activation in the VOI in the exclusion condition (*M* = 0.26, *SD* = 0.43) than in the inclusion condition [*M* = -0.09, *SD* = 0.38, *t*(21) = 4.278, *p* = 0.000, *d* = 0.86, see **Figure 6**]. Importantly, the difference in the activity between exclusion and inclusion condition was not found for the anxiety cues [*t*(21) = -0.165, *p* = 0.870]. No other effects were significant (all *p* > 0.05).

##### Hippocampus

A significant smell × condition interaction (*F* = 6.786, *p* = 0.017) emerged in the hippocampal region (Peak MNI coordinate: 32, -38, -10, see **Figure 6**). *Post hoc* analyses indicated that under the anxiety smell, the difference between the relative inhibition in the hippocampus in the exclusion condition (*M* = -0.15, *SD* = 0.34), compared to the inclusion condition (*M* = 0.02, *SD* = 0.26) was significant [*t*(21) = -2.206, *p* = 0.039, *d* = 0.56 see **Figure 6**]. Under the control smell, the activity in the hippocampus was enhanced in exclusion (*M* = 0.08, *SD* = 0.33), whereas it was inhibited in the inclusion condition [*M* = -0.07, *SD* = 0.20, *t*(21) = 2.476, *p* = 0.022, *d* = 0.55 see **Figure 6**]. Additionally, in the exclusion condition, there was a significant difference between the inhibition in the hippocampus under the smell of anxiety (*M* = -0.15, *SD* = 0.34) and its activity under the chemosensory control smell [*M* = 0.08, *SD* = 0.33; *t*(21) = -2.075, *p* = 0.05, *d* = 0.69, see **Figure 6**]. The other differences were not significant (all *p* > 0.05).

##### Middle temporal gyrus/superior temporal gyrus

A significant smell × condition interaction (*F* = 6.045, *p* = 0.023) and a significant main effect of condition (*F* = 12.505, *p* = 0.002) were observed in the VOI encompassing the right middle temporal gyrus and the superior temporal gyrus (Peak MNI coordinate: 62, -40, 2, see **Figure 6**). As for the main effect of condition, the participants in the exclusion condition showed inhibition in the volume (*M* = -0.04, *SD* = 0.50) whereas in the inclusion condition they showed activity in this region (*M* = 0.24, *SD* = 0.42, *t*(21) = -3.530, *p* = 0.002, *d* = 0.60]. *Post hoc* comparisons disentangling the interaction revealed that under the anxiety smell, the difference between the relative inhibition in the region in the exclusion condition (*M* = -0.32, *SD* = 0.66) as compared to the activity in the inclusion condition (*M* = 0.21, *SD* = 0.46) was significant [*t*(21) = -3.702, *p* = 0.001, *d* = 0.93, see **Figure 6**]. Moreover, in the exclusion condition, there was a significant difference between the relative inhibition in the area under the smell of anxiety (*M* = -0.32, *SD* = 0.66) and the activity under the chemosensory control cues [*M* = 0.24, *SD* = 0.84; *t*(21) = -2.344, *p* = 0.029, *d* = 0.74, see **Figure 6**]. The other differences were not significant (all *p* > 0.05).

##### Inferior frontal gyrus

A significant smell × condition interaction (*F* = 11.729, *p* = 0.003) and a significant main effect of condition (*F* = 19.928, *p* = 0.000) were identified in the Inferior Frontal Gyrus (Peak MNI coordinate: -46, 26, 18, see **Figure 6**). Across chemosensory conditions, during exclusion condition the participants showed inhibition in the IFG (*M* = -0.12, *SD* = 0.28) whereas in the inclusion condition they showed activity in this region [*M* = 0.08, *SD* = 0.24, *t*(21) = -4.438, *p* = 0.000, *d* = 0.77]. Under the anxiety smell (but not sports), the difference between the inhibition in IFG in the exclusion condition (*M* = -0.22, *SD* = 0.35) as compared to the activity in the inclusion condition (*M* = 0.15, *SD* = 0.34) was significant [*t*(21) = -5.887, *p* = 0.000, *d* = 1.1, see **Figure 6**]. No other significant differences emerged (all *p* > 0.05).

## Discussion

The central question of the current study was focused on the neuronal implications of chemosensory anxiety signals in the context of social exclusion: do they deepen the negative experience of ostracism or alleviate it? We implemented a widely used paradigm to study social exclusion – Cyberball — while exposing participants to chemosensory cues signaling anxiety (versus control cues). The results revealed that exposure to anxiety cues: (1) modulates the activity in the brain regions involved in processing of negative experience of social exclusion and (2) down-regulates the neuronal areas involved in socio-emotional cognition. These results suggest that chemosensory anxiety signals might diminish the experience of social exclusion and facilitate withdrawal from others in the context of a stressful social situation.

### Neural Responses to Ostracism under Chemosensory Anxiety Cues

Our results extend conclusions of previous studies in the area of chemosensory communication, by showing the association between the exposure to chemosensory anxiety signals and modulation of neural responses during an actual threatening situation of social ostracism. Specifically, during the episode of social rejection the presentation of chemosensory anxiety was not associated with increased activation of the regions previously implied in social rejection (e.g., Eisenberger et al., 2003; Eisenberger, 2012), and also observed in our control chemosensory condition (i.e., anterior cingulate, medial frontal gyrus, orbitofrontal cortices). The lack of activation in these regions, known to be a part of the pain matrix (Eisenberger et al., 2003; Eisenberger, 2012; although a recent meta-analysis by Cacioppo et al., 2013, challenged this perspective arguing that neural correlates of social pain are more complex than claimed by those earlier studies), might imply that chemosensory anxiety moderates the experience of social rejection observed in the control situation.

Moreover, simultaneous deactivations in the brain regions involved in memory (hippocampus), social cognition (middle temporal gyrus, superior temporal gyrus) and salience processing (inferior frontal gyrus) might suggest that successful communication of chemosensory anxiety may be linked to enhancing the preparation of the individual to tackle a stressful episode (e.g., in line with Mujica-Parodi et al., 2009; Zhou and Chen, 2009). Previous studies demonstrated that anxiety signals are driven by the activation of the SAM system (de Groot et al., 2015), which plays a role in the initiation of the fight/flight response. If this state is at least partially communicated to the sweat recipients, it can be presumed that anxiety signals in the context of a distressing social situation may promote the emergence of mechanisms helping to address the hazardous scene e.g., via promotion of distance from the emotional state, or withdrawal from the rejection scenes (also see Koenigsberg et al., 2010; Premkumar, 2012). Accordingly, in the current experiment, upon presentation of the anxiety signals during exclusion, the relative deactivation of the hippocampal area, the region involved in memory, especially encoding processes (Brewer et al., 1998; Wagner et al., 1998), suggests diminished imprinting of negative events into the long term-memory. Given that the opposite pattern was observed in the control chemosensory condition, i.e., rejection led to stronger activity in the region, it appears that chemosensory anxiety plays a role in modulating this process in the context of a challenging situation such as ostracism. Correspondingly, the inhibition of the region encompassing middle temporal gyrus and superior temporal gyrus during the socially distressing episode under anxiety chemosensory cues implies decreased processing of social cues and diminished inclination for “theory of mind” or mentalizing processes, which could be related to increased detachment from the social experience altogether. Similarly, the modulation of inferior frontal gyrus activity in the anxiety (but not in the control chemosensory condition) suggests an influence of anxiety signals on salience processing: while being in a social situation with others (inclusion) the anxiety cues might add salience or arousal to the situation, as the chemosensory input may be perceived as an alarm signal. However, during dissociation from that situation (exclusion), the relative deactivation of the inferior frontal gyrus might be a result of the potential withdrawal from “social threat” signaled by anxiety sweat, which in turn lowers the inferior frontal gyrus activity.

Although, these results might appear counterintuitive in light of findings that anxiety cues enhance the salience of fear-related stimuli and the activation of the socio-emotional regions (Mujica-Parodi et al., 2009; Prehn-Kristensen et al., 2009) they suggest that anxiety signals in an actual, distressing context might override other functions and emphasize the primary role of the chemosensory “alarm” signals in the animal kingdom which is to initiate the organisms’ withdrawal behavior from the situation appraised as threatening (e.g., Suh et al., 2004). This interpretation is also in line with a strong body of research showing that the participants exposed to sweat collected from stress-inducing social situation show other signs of preparedness for threat such as improved cognitive performance (Chen et al., 2006), enhanced sensory acquisition (sensory intake, increased visual field size, de Groot et al., 2012) and activation of the withdrawal systems (Prehn et al., 2006; Pause et al., 2009). It should be noted that the withdrawal in light of anxiety signals can be considered a positive outcome, as it facilitates the possible threatening stimulus that leads the sender of the signal to be alarmed in the first place. Future studies should investigate the interaction between the anxiety signals, processing of salience and withdrawal motivation. They should also further decode the mechanisms by which the anxiety signals might promote disconnection from the difficult social situation (e.g., what emotional or physiological tactics are employed by those exposed to the chemosensory cues). This is particularly important, because, given that the currently observed neural regions are involved in a wide range of processes, beyond social cognition and salience, it cannot be precluded, that other processes contribute to the observed results as well.

Lastly, we did not observe any gender effects in the current research, which suggests that the exposure to anxiety signals does not influence neural responses to social exclusion, as a function of gender. Although this is consistent with research showing that both males and females show similar neural activation patterns in response to the olfactory samples of male fear signals (Radulescu and Mujica-Parodi, 2013), studies with larger samples of men and women, designed to test for gender effects specifically are encouraged, to further explore possible differences in experience of social exclusion under influence of chemosensory cues in men and women.

### Limitations

Although the commonly implemented Cyberball task offers a relatively high ecological validity, it suffers from several limitations when used in the fMRI scanner. Particularly problematic are: (1) the exclusion condition follows the inclusion condition and thus there is a risk of the neural responses being a result of expectancy violation (Somerville et al., 2006), and (2) the length of the blocks leads to a less-than-optimal signal-to-noise ratio. With regards to the current experiment, the choice of a within-subject design, although beneficial for measuring intraindividual variability, might have reduced the experience of social exclusion in the second round of the game (as the repeated rejection trial following inclusion trial appears less realistic). It has to be noted, however, that the order of odor presentation was counterbalanced across participants, such that a “lack of belief in the second exclusion” problem should not occur for different odors. Moreover, several studies have indicated that even a lack of belief in the cover story or knowledge of the exclusion being simulated by the computer is nevertheless associated with the automatic response typical for ostracism (Zadro et al., 2004; Sebastian et al., 2011, also in line with Williams, 2007). Further, the inclusion of a control chemosensory condition, in which non-social cues were presented, would be beneficial to drawing the conclusions regarding the social chemosensory nature of the effects. Lastly, the visual depiction in the differential contrast sports exclusion > sports inclusion (**Figure 5**) suggests a possible motion artifact in the third panel. However, given that the motion parameters were included into our design (which should minimize such effects) and the extent of this cluster is in large part in the gray matter (with peak MNI coordinates within Superior Occipital Gyrus), it appears that the activation is not solely artifact related.

## Conclusion

The current results suggest that successful communication of anxiety via chemosensory domain is associated with down-regulation of regions typically implied in the experience of social exclusion during social ostracism. Moreover, it suggests that chemosensory anxiety cues distance individuals from the group, by inhibiting social cognition, salience and memory formation regions during a distressing social event. Cumulatively, it is possible that the anxiety signals make it easier for the individuals to withdraw from the hazardous social situation, pointing to the communicative role of chemosensory “alarm” cues in enhancing adjustment mechanisms in light of distressing circumstances.

## Conflict of Interest Statement

The authors declare that the research was conducted in the absence of any commercial or financial relationships that could be construed as a potential conflict of interest.

## Footnotes

1 http://www.fil.ion.ucl.ac.uk/spm

2 http://nmr.mgh.harvard.edu/harvardagingbrain/People/AaronSchultz/Aarons_Scripts.html

3 www.alivelearn.net/xjview8

4 http://marsbar.sourceforge.net/

## References

[B1] Ashburner J. Friston K. J. (2005). Unified segmentation. *Neuroimage* 26 839–851. 10.1016/j.neuroimage.2005.02.018 15955494

[B2] Baumeister R. F. Leary M. R. (1995). The need to belong: desire for interpersonal attachments as a fundamental human motivation. *Psychol. Bull.* 117 497 10.1037/0033-2909.117.3.497 7777651

[B3] Beck A. T. Ward C. Mendelson M. Mock J. Erbaugh J. (1961). Depth of depression. *Arch. Gen. Psychiat.* 4 561–571. 10.1001/archpsyc.1961.01710120031004 13688369

[B4] Bowlby J. (1969). *Attachment: (Attachment and Loss Series, Vol. 1).* New York: Basic Books.

[B5] Bradley M. M. Lang P. J. (1994). Measuring emotion: the self-assessment manikin and the semantic differential. *J. Behav. Ther. Exp. Psychiat.* 25 49–59. 10.1016/0005-7916(94)90063-9 7962581

[B6] Brewer J. B. Zhao Z. Desmond J. E. Glover G. H. Gabrieli J. D. (1998). Making memories: brain activity that predicts how well visual experience will be remembered. *Science* 281 1185–1187. 10.1126/science.281.5380.1185 9712581

[B7] Brown G. E. Poirier J. F. Adrian J. C. (2004). Assessment of local predation risk: the role of subthreshold concentrations of chemical alarm cues. *Behav. Ecol.* 15 810–815. 10.1093/beheco/arh084

[B8] Cacioppo S. Frum C. Asp E. Weiss R. M. Lewis J. W. Cacioppo J. T. (2013). A quantitative meta-analysis of functional imaging studies of social rejection. *Sci. Rep.* 3 1–3. 10.1038/srep02027 PMC3761131 24002359

[B9] Carrington S. J. Bailey A. J. (2009). Are there theory of mind regions in the brain? A review of the neuroimaging literature. *Hum. Brain Mapp.* 30 2313–2335. 10.1002/hbm.20671 19034900 PMC6871093

[B10] Chen D. Katdare A. Lucas N. (2006). Chemosignals of fear enhance cognitive performance in humans. *Chem. Senses* 31 415–423. 10.1093/chemse/bjj046 16527869

[B11] Cohen J. (1988). *Statistical Power for the Social Sciences.* Hillsdale, NJ: Laurence Erlbaum and Associates.

[B12] Cox R. W. (1996). AFNI: software for analysis and visualization of functional magnetic resonance neuroimages. *Comput. Biomed. Res.* 29 162–173. 10.1006/cbmr.1996.0014 8812068

[B13] de Groot J. H. Semin G. R. Smeets M. A. (2014). I can see, hear, and smell your fear: comparing olfactory and audiovisual media in fear communication. *J. Exp. Psychol. Gen.* 143 825 10.1037/a0033731 23855495

[B14] de Groot J. H. Smeets M. A. Kaldewaij A. Duijndam M. J. Semin G. R. (2012). Chemosignals communicate human emotions. *Psychol. Sci.* 23 1417–1424. 10.1177/0956797612445317 23019141

[B15] de Groot J. H. Smeets M. A. Semin G. R. (2015). Rapid stress system drives chemical transfer of fear from sender to receiver. *PLoS ONE* 10:e0118211 10.1371/journal.pone.0118211 PMC4344325 25723720

[B16] Doty R. L. (1981). Olfactory communication in humans. *Chem. Senses* 6 351–376. 10.1093/chemse/6.4.351

[B17] Eickhoff S. Stephan K. Mohlberg H. (2005). A new SPM toolbox for combining probabilistic cytoarchitectonic maps and functional imaging data. *Neuroimage* 25 1325–1335. 10.1016/j.neuroimage.2004.12.034 15850749

[B18] Eisenberger N. I. (2012). The pain of social disconnection: examining the shared neural underpinnings of physical and social pain. *Nat. Rev. Neurosci.* 13 421–434. 10.1038/nrn3231 22551663

[B19] Eisenberger N. I. Lieberman M. D. Williams K. D. (2003). Does rejection hurt? An fMRI study of social exclusion. *Science* 302 290–292. 14551436 10.1126/science.1089134

[B20] Ekman P. Friesen W. V. (1975). *Unmasking the Face: A Guide to Recognizing Emotions from Facial Clues.* Englewood Cliffs, NJ: Prentice-Hall.

[B21] First M. B. Spitzer R. L. Gibbon M. Williams J. B. (1995). *Structured Clinical Interview for DSM-IV Axis I Disorders (SCID).* New York: New York State Psychiatric Institute, Biometrics Research.

[B22] Freiherr J. Gordon A. R. Alden E. C. Ponting A. L. Hernandez M. F. Boesveldt S. (2012). The 40-item monell extended sniffin’sticks identification test (MONEX-40). *J. Neurosci. Methods* 205 10–16. 10.1016/j.jneumeth.2011.12.004 22200409 PMC3623611

[B23] Hampshire A. Chamberlain S. R. Monti M. M. Duncan J. Owen A. M. (2010). The role of the right inferior frontal gyrus: inhibition and attentional control. *Neuroimage* 50 1313–1319. 10.1016/j.neuroimage.2009.12.109 20056157 PMC2845804

[B24] Inagaki H. Kiyokawa Y. Kikusui T. Takeuchi Y. Mori Y. (2008). Enhancement of the acoustic startle reflex by an alarm pheromone in male rats. *Physiol. Behav.* 93 606–611. 10.1016/j.physbeh.2007.10.021 18061219

[B25] Koenigsberg H. W. Fan J. Ochsner K. N. Liu X. Guise K. Pizzarello S. (2010). Neural correlates of using distancing to regulate emotional responses to social situations. *Neuropsychologia* 48 1813–1822. 10.1016/j.neuropsychologia.2010.03.002 20226799 PMC2905649

[B26] Kohn N. Eickhoff S. B. Scheller M. Laird A. R. Fox P. T. Habel U. (2014). Neural network of cognitive emotion regulation—an ALE meta-analysis and MACM analysis. *Neuroimage* 87 345–355. 10.1016/j.neuroimage.2013.11.001 24220041 PMC4801480

[B27] Kohn N. Falkenberg I. Kellermann T. Eickhoff S. B. Gur R. C. Habel U. (2013). Neural correlates of effective and ineffective mood induction. *Soc. Cogn. Affect. Neurosci.* 9 864–872. 10.1093/scan/nst055 23576810 PMC4040099

[B28] Leveroni C. L. Seidenberg M. Mayer A. R. Mead L. A. Binder J. R. Rao S. M. (2000). Neural systems underlying the recognition of familiar and newly learned faces. *J. Neurosci.* 20 878–886. 10632617 10.1523/JNEUROSCI.20-02-00878.2000 PMC6772415

[B29] Morris R. G. M. (2007). “Theories of hippocampal function,” in *The Hippocampus Book*, eds Andersen P. Morris R. Amaral D. Bliss T. O’Keefe J. (Oxford: Oxford University Press), 581–713.

[B30] Mujica-Parodi L. R. Strey H. H. Frederick B. Savoy R. Cox D. Botanov Y. (2009). Chemosensory cues to conspecific emotional stress activate amygdala in humans. *PLoS ONE* 4:e6415 10.1371/journal.pone.0006415 PMC2713432 19641623

[B31] Oldfield R. C. (1971). The assessment and analysis of handedness: the Edinburgh inventory. *Neuropsycholgia* 9 97–113. 10.1016/0028-3932(71)90067-4 5146491

[B32] Pause B. M. (2012). Processing of body odor signals by the human brain. *Chemosens. Percept.* 5 55–63. 10.1007/s12078-011-9108-2 22448299 PMC3309140

[B33] Pause B. M. Adolph D. Prehn-Kristensen A. Ferstl R. (2009). Startle response potentiation to chemosensory anxiety signals in socially anxious individuals. *Int. J. Psychophysiol.* 74 88–92. 10.1016/j.ijpsycho.2009.07.008 19666058

[B34] Prehn A. Ohrt A. Sojka B. Ferstl R. Pause B. M. (2006). Chemosensory anxiety signals augment the startle reflex in humans. *Neurosci. Lett.* 394 127–130. 10.1016/j.neulet.2005.10.012 16257486

[B35] Prehn-Kristensen A. Wiesner C. Ole Bergmann T. O. Wolf S. Jansen O. Maximilian Mehdorn H. (2009). Induction of empathy by the smell of anxiety. *PLoS ONE* 4:e5987 10.1371/journal.pone.0005987 PMC2695008 19551135

[B36] Premkumar P. (2012). Are you being rejected or excluded? Insights from neuroimaging studies using different rejection paradigms. *Clin. Psychopharmacol. Neurosci.* 10 144–154. 10.9758/cpn.2012.10.3.144 23430682 PMC3569164

[B37] Radulescu A. R. Mujica-Parodi L. R. (2013). Human gender differences in the perception of conspecific alarm chemosensory cues. *PLoS ONE* 8:e68485 10.1371/journal.pone.0068485 PMC3722227 23894310

[B38] Sebastian C. L. Tan G. C. Roiser J. P. Viding E. Dumontheil I. Blakemore S. J. (2011). Developmental influences on the neural bases of responses to social rejection: implications of social neuroscience for education. *Neuroimage* 57 686–694. 10.1016/j.neuroimage.2010.09.063 20923708

[B39] Somerville L. H. Heatherton T. F. Kelley W. M. (2006). Anterior cingulate cortex responds differentially to expectancy violation and social rejection. *Nat. Neurosci.* 9 1007–1008. 10.1038/nn1728 16819523

[B40] Stevenson R. J. (2010). An initial evaluation of the functions of human olfaction. *Chem. Senses* 35 3–20. 10.1093/chemse/bjp083 19942579

[B41] Suh G. S. Wong A. M. Hergarden A. C. Wang J. W. Simon A. F. Benzer S. (2004). A single population of olfactory sensory neurons mediates an innate avoidance behaviour in Drosophila. *Nature* 431 854–859. 10.1038/nature02980 15372051

[B42] Tempini M. G. Price C. J. Josephs O. Vandenberghe R. Cappa S. F. Kapur N. (1998). The neural systems sustaining face and proper name processing. *Brain* 121 2103–2118. 10.1093/brain/121.11.2103 9827770

[B43] Wagner A. D. Schacter D. L. Rotte M. Koutstaal W. Maril A. Dale A. M. (1998). Building memories: remembering and forgetting of verbal experiences as predicted by brain activity. *Science* 281 1188–1191. 10.1126/science.281.5380.1188 9712582

[B44] Williams K. D. (2007). Ostracism. *Annu. Rev. Psychol.* 58 425–452. 10.1146/annurev.psych.58.110405.085641 16968209

[B45] Williams K. D. Cheung C. K. Choi W. (2000). Cyberostracism: effects of being ignored over the Internet. *J. Pers. Soc. Psychol.* 79 748 10.1037/0022-3514.79.5.748 11079239

[B46] Williams K. D. Govan C. L. Croker V. Tynan D. Cruickshank M. Lam A. (2002). Investigations into differences between social and cyber ostracism. *Group Dyn Theor. Res.* 6 65–77. 10.1037/1089-2699.6.1.65

[B47] Williams K. D. Jarvis B. (2006). Cyberball: a program for use in research on interpersonal ostracism and acceptance. *Behav. Res. Methods* 38 174–180. 10.3758/BF03192765 16817529

[B48] Zadro L. Williams K. D. Richardson R. (2004). How low can you go? Ostracism by a computer is sufficient to lower self-reported levels of belonging, control, self-esteem, and meaningful existence. *J. Exp. Soc. Psychol.* 40 560–567.

[B49] Zhou W. Chen D. (2009). Fear-related chemosignals modulate recognition of fear in ambiguous facial expressions. *Psychol. Sci.* 20 177–183. 10.1111/j.1467-9280.2009.02263.x 19170944

