# Supplementary figures and images for: Chemosensory anxiety cues moderate the experience of social exclusion – an fMRI investigation with Cyberball
Source: Front Psychol. 2015 Oct 9;6:1475. doi: 10.3389/fpsyg.2015.01475 (PMC4599064; doi:10.3389/fpsyg.2015.01475)

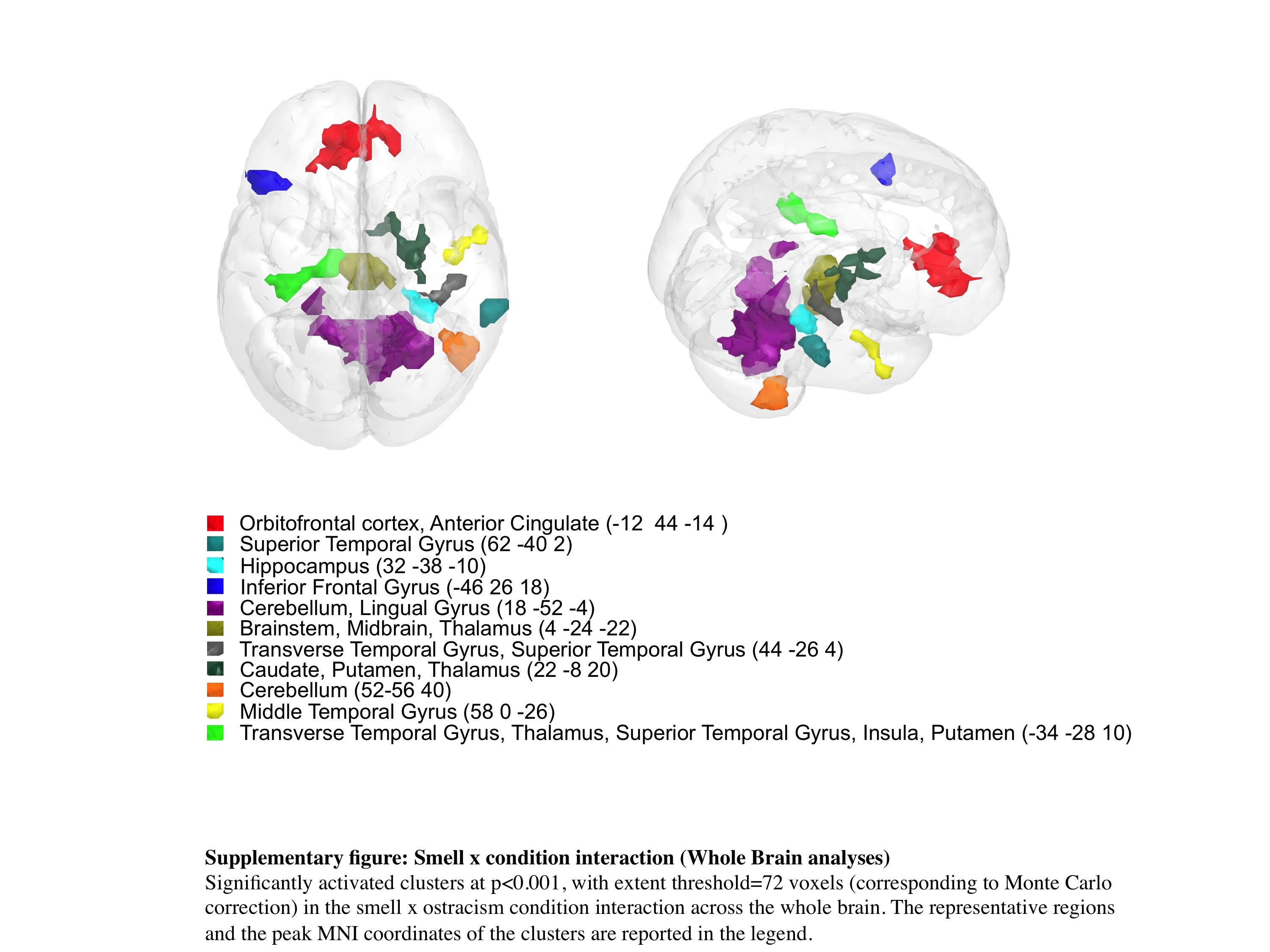

Supplement: Supplementary file 1 [file Image_1.JPEG]
